# Presence of On-Target Resistant Mutation in Pre-Treatment Samples of ALK Fusion Gene Positive Lung Cancer Patients

**DOI:** 10.3390/cancers17071090

**Published:** 2025-03-25

**Authors:** Weiting Li, Fenneke Zwierenga, Katarina D. Andini, Justyna M. Bucher, Frank Scherpen, T. Jeroen N. Hiltermann, Harry J. M. Groen, Anthonie J. van der Wekken, Klaas Kok, Anke van den Berg

**Affiliations:** 1 Department of Pathology and Medical Biology, University of Groningen, University Medical Centre Groningen, 9713 GZ Groningen, The Netherlands; w.li@umcg.nl (W.L.); f.j.g.scherpen@umcg.nl (F.S.); 2 Department of Pulmonary Diseases, University of Groningen, University Medical Centre Groningen, 9713 GZ Groningen, The Netherlands; f.zwierenga@umcg.nl (F.Z.); t.j.n.hiltermann@umcg.nl (T.J.N.H.); h.j.m.groen@umcg.nl (H.J.M.G.); a.j.van.der.wekken@umcg.nl (A.J.v.d.W.); 3 Department of Genetics, University of Groningen, University Medical Centre Groningen, 9700 RB Groningen, The Netherlands; k.d.andini@umcg.nl (K.D.A.); justyna.bucher@gmail.com (J.M.B.)

**Keywords:** ALK, NSCLC, resistance, mutation, ddPCR

## Abstract

A subset of ALK+ non-small cell lung cancer (NSCLC) patients relapse on ALK inhibitor (ALKi) treatment due to on-target resistance mutations affecting the tyrosine kinase domain. Objective: In this study, we investigated the presence of minor resistant clones in pre-treatment tissue samples and assessed their predictive value for subsequent resistance mechanisms. Methods: Using the highly sensitive digital droplet (dd)PCR technique, we analyzed 40 tissue samples obtained from 17 patients who had developed on-target resistance mutations after receiving ALKi between 2013 and 2022. We focused on 10 on-target ALKi resistant mutations identified in our patient cohort. Results: Fifteen ALKi resistance mutations were detected in 13 samples from 11/17 patients. Among these, four mutations were observed as resistance mutations in follow-up biopsies taken after first or subsequent lines of ALKi. Comparison of the test results from two subsequent biopsies, before and directly after therapy, revealed presence of the resistance mutation identified upon relapse in the pre-treatment sample of three cases that were all taken from the same tumor location. In six cases taken from different tumor locations, the resistant mutations were not found in the pre-treatment sample. Conclusions: By using the highly sensitive ddPCR approach, we detected minor clones with on-target resistant mutations in both treatment-naive and relapse biopsies from ALK-positive NSCLC patients. The predictive value of these mutations as the potential resistance-causing mechanism was limited to relapses occurring at the same tumor location as the pre-treatment sample.

## Simple Summary

Lung cancer patients with an anaplastic lymphoma kinase (ALK)-fusion can be treated effectively with ALK inhibitors. However, patients generally relapse within one to two years. In a proportion of these patients, the resistance is caused by mutations in the kinase domain of the ALK-fusion gene. Our aim was to assess if the presence of ALK-resistance associated mutations in the tumor tissue before the start of first or subsequent lines of ALK-inhibitor therapy was predictive for the resistance mechanism. We analyzed 17 patients presenting with proven on-target ALK resistant mutations in at least one of the follow-up samples using the highly sensitive ddPCR approach. We only detected minor clones with ALK resistance mutations evolving into the dominant resistant mutation in cases where the relapses occurred at the same tumor location as the pre-treatment sample. Our study provides novel insights into the heterogeneity of resistance mechanisms occurring in different tumor locations.

## 1. Introduction

The anaplastic lymphoma kinase (ALK) receptor was identified as a driver gene in non-small cell lung cancer (NSCLC) in 2007 [1,2,3,4]. Activation of the tyrosine kinase domain of ALK occurs as result of a chromosomal rearrangement, such as inversions or translocations, leading to the formation of an ALK fusion gene. The most common fusion partner is echinoderm microtubule-associated protein-like 4 (EML4) [1]. As a result of the fusion, the TKI domain of ALK is placed under control of the EML4 gene promotor. In addition, the coiled coil domain of EML4 contributes to dimerization and activation of ALK [5]. Following this discovery, multiple ALK inhibitors (ALKi) have been developed, and their efficacy has been assessed in lung cancer patients. In 2011, crizotinib was approved by the FDA as the first-line targeted therapy in ALK-positive NSCLC [6,7]. While the initial response to crizotinib is typically favorable among ALK-positive patients, relapse commonly occurs within one to two years [8,9,10]. In approximately 20% of patients who received ALKi therapy, secondary on-target point mutations resulting in resistance are observed in the kinase domain [11]. In the other patients, resistance is caused by other so-called off-target resistance mechanisms. Secondary on-target ALK point mutations include p.(L1196M), p.(G1269A), p(C1156Y), p.(G1202R), p.(I1171T/N/S), p.(S1206C/Y), p(E1210K), p.(L1152P/R), p.(V1180L), p.(I1151T), and p.(F1174C) [12]. Causal links between several of these secondary point mutations and ALKi resistance have been shown in cell line models and clinical studies [11]. The presence of on-target mutations prompts the development of additional ALKi that may overcome the observed resistance, such as ceritinib [13], brigatinib [14], and lorlatinib [15]. In 2017, alectinib was approved as first-line treatment for ALK-fusion positive NSCLC patients in Europe, as well as for patients experiencing relapse after crizotinib treatment [11,12]. Unfortunately, even with these advancements, patients inevitably still develop resistance to treatment [16].

The on-target mutations that cause resistance to ALKi can either be pre-existing as minor clones or be acquired during therapy. This phenomenon has previously been studied for the p.(T790M) on-target mutation in NSCLC patients with activating mutations in the epidermal growth factor receptor (EGFR) [17,18,19,20]. Currently, the prevalence of pre-existing on-target resistance mutations in ALK fusion-positive NSCLC remains unclear. So far, one study has reported the presence of an ALK p.(L1196M) mutation in one out of three tissue samples taken prior to crizotinib treatment of ALK fusion positive patients [18]. In this study we explored the presence of minor clones with pre-existing resistance mutations in TKI-naïve and relapse samples. When present, these clones with TKI-resistance-causing mutations could have a negative effect on progression-free survival. Thus, their detection could be of clinical relevance and potentially impact selection of the most optimal TKI for treatment.

## 2. Materials and Methods

### 2.1. Patient Selection, Tumor and Control Samples

From April 2013 until March 2023, we identified 17 patients with acquired on-target resistance mutations from our previously diagnosed and ALKi-treated ALK fusion gene positive patients (*n* = 96). An overview of the results of the diagnostics tests, estimated tumor cell content, and method of biopsy procurement are summarized in Supplementary Table S1. Clinical data were retrieved from the medical records of the University Medical Center Groningen, such as patient history (gender, age, smoking status, tumor histology and molecular profile, initial stage of disease) and treatment data (Table 1). All ALK fusion gene and mutation tests were performed according to standard procedures at the time of diagnosis in our ISO15189 certified diagnostic lab (Supplementary Table S1). Survival data were determined according to RECIST 1.1 [21]. Overall survival (OS) was defined as time from diagnosis until death or date of cut off at 1st March 2023. Progression free survival (PFS) per line of ALKi was defined as time from anti-tumor treatment to detection of progressive disease or death or date of cutoff. Duration of treatment (DoT) was defined as the time between start and end of treatment. On-target ALKi resistant mutations following first treatment line (referred to as sample 1) or subsequent treatment lines (referred to as samples 2, 3, or 4) were analyzed by ddPCR for the respective resistance mutations. The patients whose data and samples were included in this study had given informed consent to the Oncological Life Study (OncoLifeS) data-biobank (https://www.umcg.nl/-/oncolifes, accessed on 20 March 2025). The OncoLifeS data-biobank received approval from the medical center’s medical ethical committee [22].

DNA samples were retrieved from the molecular diagnostics archive for all available samples of the ALKi resistance mutation-positive patients, as well as control formalin fixed paraffin embedded (FFPE) tumor samples. Details on the isolation procedures are given in the Supplemental Data and Supplementary Tables S1 and S2. DNA extraction was performed from macrodissected tissue areas containing a tumor cell percentage of 20% or higher, as indicated by an accredited pathologist (estimates of the enriched tumor cell content are given in Supplementary Table S1). Control FFPE samples were selected from the diagnostic archive based on being isolated in the same weeks as the ALK patient samples (For details see Supplemental Table S2). Overall, 11 of the 17 control cases had a known driver gene mutation (no ALK fusion gene) with a mutant allele frequency ranging between 22% and 76% and a median of 34%. This indicates presence of sufficient tumor cells in the control FFPE blocks. The control FFPE samples were used to determine the cutoff value for each ddPCR assay. As our previous study using ddPCR on FFPE samples showed a higher number of positive droplets for DNA samples from aged as compared to freshly prepared FFPE blocks [20], we checked the time interval between the generation of FFPE blocks and the date of DNA isolation for FFPE blocks included in this study. All DNA isolations were conducted within one month of biopsy collection, ensuring a consistent age of the FFPE blocks at the time of DNA isolation for controls and patient samples. High quality genomic DNA samples isolated from the white blood cells (WBC) of 10 healthy controls were used to test the specificity of the mutant and wild type probes in the droplet digital PCR (ddPCR).

### 2.2. Generation of Positive Controls for ddPCR

Although the ddPCR assays were commercially available, positive control DNA samples were not readily accessible. To address this issue, we generated DNA fragments of 200–250 nucleotides containing 7 of the 10 selected ALK mutations detected in the fusion gene-positive patients (see Supplemental Methods, and Tables S3 and S4). Presence of the mutation in the positive controls was confirmed by Sanger sequencing (Supplementary Table S3). For the p.(S1206A), p.(E1210K), and p.(F1174L) mutations, gBlocks containing the specific mutation were ordered from IDT (IDT, Leuven, Belgium) (Supplementary Table S4). All positive control fragments were mixed with fragments lacking mutations to achieve a variant allele frequency (VAF) of approximately 15%. The positive controls were used to determine the optimal annealing temperature for each assay.

### 2.3. Droplet Digital (dd)PCR

The ddPCR assays were procured from Bio-Rad (Bio-Rad Laboratories BV, Veenendaal, The Netherlands) (Supplementary Table S5). Reactions were conducted in accordance with the manufacturer’s protocol. In brief, amplification reactions were carried out in a volume of 20 µL, containing fluorescently labeled mutation-specific (FAM) and wild-type-specific (HEX) probes, as previously described (Supplementary Figure S3) [20]. Droplets were generated by the ddPCR generator (Bio-Rad Laboratories BV, Veenendaal, The Netherlands). Fluorescence signals from individual droplets were detected by the QX200 droplet reader (Bio-Rad Laboratories BV, Veenendaal, The Netherlands) and analyzed using QuantaSoft Analysis Pro software (version 1.0.596, Bio-Rad). For each assay, we defined the optimal annealing temperature to discriminate between wild and mutant droplets using the synthetic control DNA. Next, we assessed the specificity of the mutant probes in two ways. First, we tested each ddPCR assay on genomic DNA isolated from WBC. Next, we analyzed all 10 synthetic positive controls to determine cross-reactivity, which is critical specifically for ALKi resistance mutations at the same or closely flanking genomic positions. Each DNA sample was analyzed in at least two independent experiments and in two wells per experiment. We aimed to achieve a minimum of 6000 filled droplets for each sample (sum of all wells). All samples were analyzed for up to 10 mutations, depending on the available amount of DNA. Positive and negative DNA control samples, along with a DNA-free sample, were included in each experiment. The number of WT alleles was calculated by the formula: (WT containing droplets) × (LN(1 − (WT containing droplets)/(total number of generated droplets))). This formula transformed the number of WT droplets into WT alleles by correction for the number of droplets containing two alleles. The variant allele frequency was then calculated by the formula (variant alleles × 100)/(wild type + variant alleles).

### 2.4. Statistical Analysis

We applied two criteria to consider samples to be positive for a specific mutation. The first criterion required a total of at least six FAM (mutant)-positive droplets across all analyzed wells. The second criterion involved mutant allele frequencies (MAF) that were outliers based on a cut-off value. This value was based on the Grubbs critical value, as determined on the control FFPE samples with an alpha of 0.05. Samples meeting both criteria were classified as positive for the tested ALKi resistant mutation. Our chosen criteria, requiring a minimum of six mutant droplets and aiming to analyze at least 6000 filled droplets, provided a MAF detection sensitivity of 0.1% or better.

## 3. Results

### 3.1. Patient Selection and Clinical Characteristics

**Figure 1:**
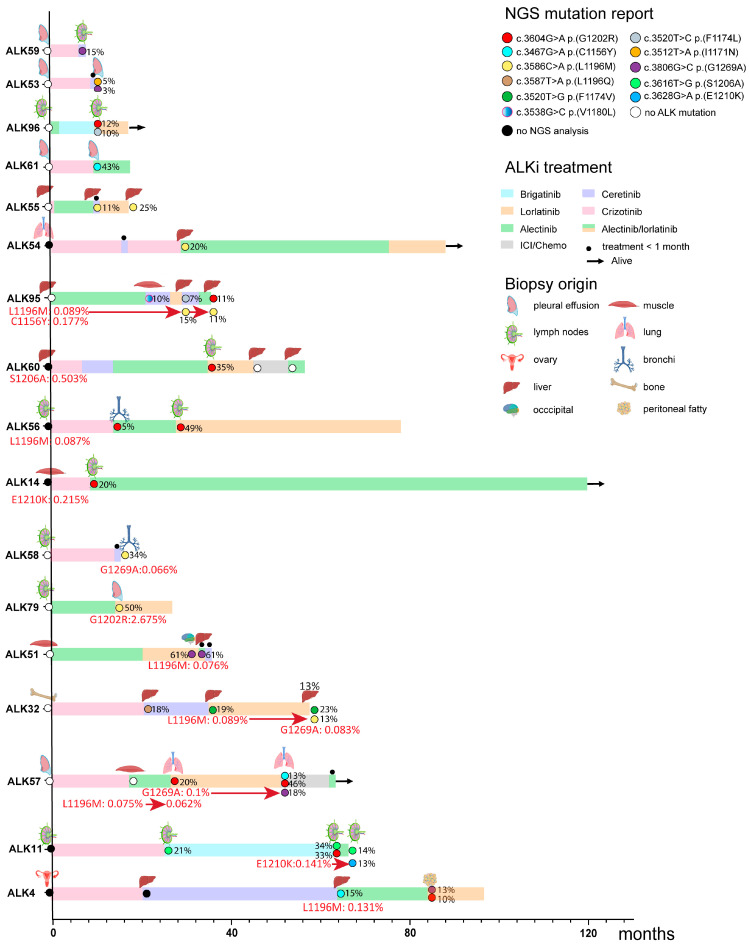
Overview of the ALK patients, including treatment, tumor locations, and mutations. Patients are sorted based on having no on-target mutations (upper 6), presence of on-target mutations in pre-ALKi treatment sample (next 4), and on-target mutations in relapse biopsies (final 7). For each patient, the ALKi treatment is indicated by the colored bar, with the length of the bar indicative of the length of the ALKi treatment. Treatments that lasted less than a month are indicated by a small black circle above the bar. On-target TKI resistance mutations identified by diagnostic procedures are indicated by a colored circle. White and black circles indicate biopsies without an on-target ALK mutation or not analyzed by NGS, respectively. Small icons above the bars indicate the origin of the biopsies. MAFs above threshold as detected by ddPCR are indicated in red text. Arrows indicate mutations detected as minor clones in pre-ALKi that were subsequently found as resistance mutations in a diagnostic NGS test.

A total of 96 patients were diagnosed with an ALK fusion-positive NSCLC at the UMCG between 2013 and 2022. Seventeen patients (18%) had an acquired on-target resistant mutation after ALKi treatment (Figure 1). Table 1 provides an overview of the clinical characteristics of these patients. At initial diagnosis, four patients presented with stage IVA disease and ten with stage IVB. Two patients initially diagnosed with stage IIIA NSCLC progressed rapidly to stage IV before starting regular treatment. Consequently, they were only treated with ALKi therapy. One patient was initially diagnosed with stage IIIB NSCLC, but curative treatment was not possible due to the tumor location. Nine patients harbored an EML4:ALK fusion gene (assessed by Nanostring or Archer), while the fusion partner was unknown in the remaining cases (ALK-positivity assessed by FISH or IHC). The presence of on-target ALKi resistant mutations upon progression following first line or subsequent lines of ALKi was assessed through routine molecular testing using targeted next generation sequencing (NGS). The ALK mutations investigated in our study were selected based on the on-target ALKi resistance mutations observed in our cohort of 17 patients. An overview of the total number of samples available after each line of TKI for these 17 patients is shown in Supplementary Figure S2.

### 3.2. Positive Controls and Cut-Off Values per Assay

**Figure 2:**
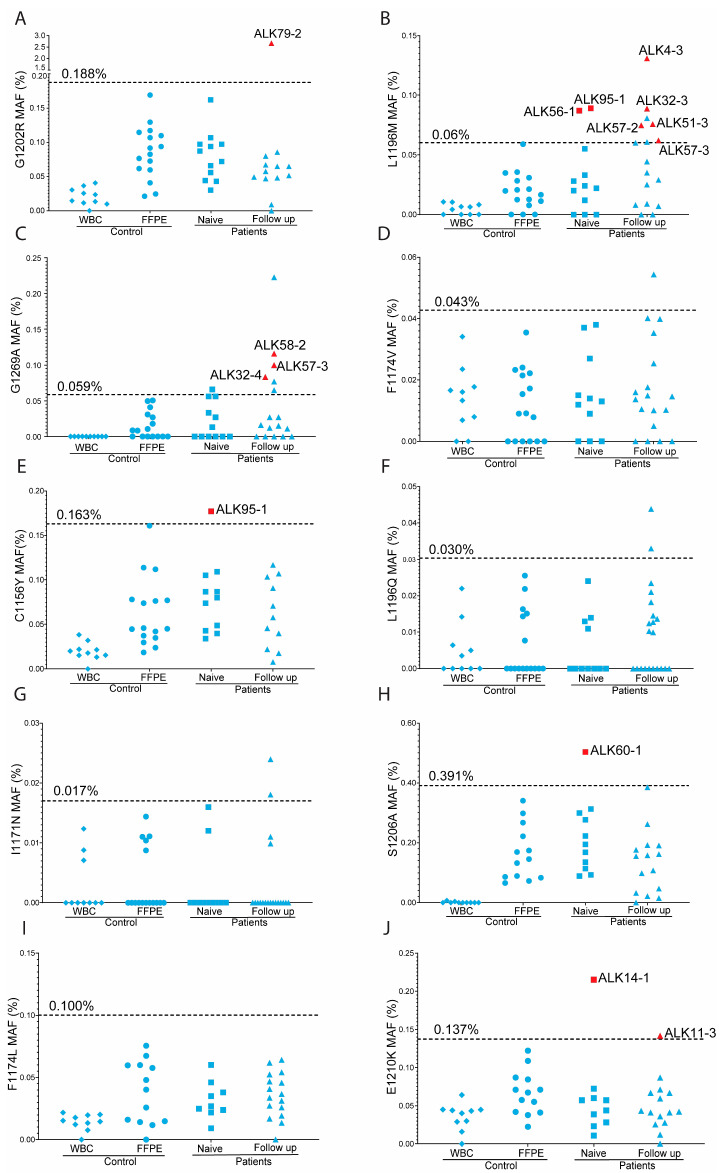
Overview of ddPCR results of controls and patients for each mutation. Horizontal dashed lines show the cut off value as determined by the Grubbs test for the control FFPE samples. The cut-off value is indicated above the dashed line. Each panel represents the data for one variant as indicated on the y-axis: (**A**) G1202R; (**B**) L1196M; (**C**) G1269A; (**D**) F1174V; (**E**) C1156Y; (**F**) L1196Q; (**G**) I1171N; (**H**) S1206A; (**I**) F1174L; (**J**) E1210K. On the x-axis, the results are shown per sample type: WBC, white blood cells; FFPE, formalin fixed paraffin embedded; Naïve; FFPE samples of treatment naïve patients; Follow up, FFPE samples of relapse of TKI treated patients. Red symbols indicate positive cases. The patient ID is indicated next to the symbol in the graph. Blue symbols above the threshold indicate samples with a MAF above the cutoff, but with less than 6 mutant droplets. Variants shown in panels (**A**,**E**,**J**) are C>T mutations.

After confirmation of the presence of the 10 selected ALK mutations in the generated positive control samples, we determined the optimal annealing temperature and assessed the specificity of the mutant probes. This analysis revealed a median number of positive droplets of 1 (range 0 to 8), and a median MAF of 0 (range 0 to 0.06%). Results of additional quality control experiments, including confirmation of mutations identified by NGS during diagnostic testing and cross-reactivity of ddPCR probes, are presented in the Supplemental Data and Supplementary Table S5. Assay specific cut-off values were determined using the Grubbs critical value (Supplementary Table S6 and Figure S3), using ddPCR data obtained for our controls (Figure 2). Notably, C>T mutations exhibited higher cutoff values (three assays, ranging from 0.14% to 0.19%) compared to six out of seven non-C>T mutations (ranging from 0.02% to 0.1%), with one exception. The p.(S1206A) ddPCR assay displayed the highest cut-off value (0.39%), surpassing even the cut-off values for the C>T ddPCR assays.

### 3.3. Overview of Overall ddPCR Results

A total of 277 ddPCR tests were conducted for the patient samples (Figure 2). An overview of the ddPCR assays tested per patient and the total number of filled droplets achieved per assay are shown in Supplementary Tables S7 and S8. Of the 277 assays, 15 (5.4%) had six or more positive droplets and an MAF above the threshold (Supplementary Tables S7 and S8). Additionally, another 10 assays showed a MAF above the threshold as defined by the Grubbs criterion but had <6 mutation-positive droplets. Of note, no outliers were observed in the total of 151 assays conducted on FFPE control samples. The difference in the number of positive samples identified in patients and controls was significantly different (*p* = 0.0029, Fisher’s exact test). This further supports the positive outcome of the ddPCR tests for the samples that fulfill both criteria.

Overall, ALKi resistance mutations were detected in 13 samples originating from 11 of the 17 patients (Figure 1). Among these, four mutations were identified as resistance-causing mutations in subsequent biopsies taken after first-line or subsequent lines of ALKi treatment, while six mutations were not observed in subsequent biopsies. For the biopsies of three patients, no subsequent biopsies were available, thus precluding further assessment. In six patients, no resistance mutations were detected by ddPCR. A detailed description of the results is given below.

### 3.4. On-Target ALKi Resistant Mutations in Treatment-Naïve Samples

Five on-target ALKi resistant mutations were detected in 4/13 ALKi-naive DNA samples (31%) (Figure 1). In patient ALK95 we detected the p.(C1156Y) (MAF = 0.177%, 14/7896 FAM-positive droplets) and the p.(L1196M) (MAF = 0.089%, 16/18,034 FAM-positive droplets) mutations. The p.(L1196M) mutation was also identified in the diagnostic setting in two subsequent biopsies obtained after multiple lines of ALKi treatment. These biopsies were taken from the same tumor location as the treatment-naïve sample (liver). The p.(C1156Y) in this patient, as well as the variants found in the other three patients, i.e., p.(S1206A) in ALK60 (MAF = 0.503%, 22/4378 FAM-positive droplets), p.(L1196M) in ALK56 (MAF = 0.087%, 7/8014 FAM-positive droplets), and p.(E1210K) in ALK14 (MAF = 0.215%, 7/3259 FAM-positive droplets), were not detected in subsequent biopsies obtained after relapse, despite re-biopsies being taken from the same tumor location in two of the three patients.

### 3.5. On-Target ALKi Resistant Mutations in Relapsed Samples

We analyzed 27 samples from 17 patients obtained after one or multiple treatment lines of ALKi, but prior to the start of a next treatment line of ALKi. In total, we identified 10 mutations in 7 out of 17 patients (41%) (Figure 1). Notably, three variants observed in three patients were also detected in subsequent biopsies taken after ALKi during routine diagnostic testing as the resistance-causing mutation. The p.(L1196M) variant observed in ALK32 (MAF = 0.089%, 10/11,223) was also reported in the diagnostic setting in a follow-up biopsy (MAF = 13%) taken from the same tumor location (liver) after treatment with lorlatinib. Similarly, the p.(E1210K) detected in ALK11 (MAF = 0.141%, 13/9193) was identified in a follow-up biopsy (MAF = 13%) after alectinib treatment, obtained from the same tumor location (lymph node next to the trachea). The p.(G1269A) observed in ALK57 (MAF = 0.1%, 10/9997) was reported in a follow-up biopsy obtained from the same tumor location (lung tissue) after treatment with lorlatinib (MAF = 18%). In contrast, the p.(L1196M) mutation observed in two biopsies of ALK57 (MAF 0.075, 11/14,720) and in a biopsy of ALK4 (MAF = 0.131%, 8/6107) was not observed as a resistance mutation in subsequent biopsies during diagnostic testing. For the four mutations detected in ALK58 (p.(G1269A) with MAF 0.116%, (7/6050), ALK79 (p.(G1202R) with MAF = 2.675% (167/6241), ALK51 p.(L1196M) with MAF = 0.076% (8/10,538), and ALK32 p.(G1269A) with MAF = 0.083% (8/9609), no subsequent re-biopsies were obtained, precluding further assessment. The p.(G1269A) mutation observed in the fourth biopsy of ALK32 was also detected in two earlier biopsies taken from the same tumor location (liver), with MAFs of 0.065% (5/9609) and 0.077% (2/2608), respectively, but with <6 mutant droplets.

### 3.6. Predictive Value of Minor Clones with On-Target ALKi Resistant Mutations

Next, we analyzed how often a mutation detected by ddPCR indeed appeared as the resistance mutation after the subsequent ALKi treatment. We had eight informative sample pairs with a total of 10 mutations (Supplementary Table S9). For three pre-treatment and post-treatment biopsy pairs taken from the same location, the mutation as observed by ddPCR in the pre-treatment sample was indeed identified as the resistance mutation by NGS in the subsequent post-treatment sample taken. In one of these pairs, with two mutations, we found only one of the two to be a resistance mutation in the post-treatment sample. The PFS of these three patients was 8.2, 13.9, and 19.4, respectively. For the five pairs with pre-treatment and post-treatment biopsies obtained from different locations, the mutations observed by ddPCR were not detected as a resistance mutation by NGS in the subsequent relapse sample. 

Vice versa, 12 out of 15 resistance mutations detected by NGS in 11 samples of 9 patients were not observed in the preceding biopsy by ddPCR. In seven cases, these biopsies were retrieved from the same tissue location, and in the other five cases, they were retrieved from different locations.

## 4. Discussion

ALKi are used as standard first line therapy in ALK-fusion positive NSCLC patients. While patients initially benefit from this treatment, they will inevitably progress due to the development of resistance mechanisms. In 20–40% of all cases, resistance will develop due to on-target mutations in the ALK kinase domain [11,18,23]. In our series of 96 ALK positive cases, on-target resistance mutations were detected in the diagnostic setting in 17 cases (18%). This is slightly lower than the frequencies observed in other studies, which reported 36% and 39% of cases as having on-target resistance mutations [19,24]. Our objective was to identify minor resistant clones harboring on-target resistant mutations prior to first line or subsequent lines of ALKi therapy. In addition to the mutations observed in the molecular diagnostics, we detected 15 ALKi resistance-causing mutations by ddPCR in 13 samples from 11 patients at a MAF well below the regular detection limit of the TSO500 NGS platform for FFPE material as used in the diagnostic setting (Illumina, San Diego, CA, USA).

The ddPCR technique is widely recognized for its reliability to detect mutations present at low MAF [25,26]. In a clinical study focusing on FFPE and cfDNA samples, ddPCR demonstrated 100% concordance with routine Sanger sequencing in detecting BRAF mutations [27]. In our study, the lower limit of detection was predominantly influenced by the amount and quality of the available DNA and thus the number of filled droplets. The criteria applied in this study resulted in a sensitivity for the MAF of 0.1%. The specificity of the mutant probes as tested in WBC samples was high, with median MAF ranging from 0% to 0.04%. In general, we observed more positive droplets for C>T mutations as compared to non-C>T mutations in FFPE control samples, consistent with previous findings [28]. This affected the lower limit of detection, especially for C>T mutations. Based on our results, it is advisable for future studies to adopt this approach to ensure robust and accurate detection of mutations using ddPCR.

It is important to recognize the redundancy in on-target ALKi resistant mutations across all available ALKi. As an example, p.(L1196M) confers resistance to five different ALKi [29]. For p.(E1210K), detected in ALK14 after relapse on crizotinib, its role in resistance remains unclear, although the current literature suggests that numerous on-target ALK mutations can inhibit the action of crizotinib [30]. Some studies suggest that the p.(G1202R) variant may be an on-target resistant mutation associated with alectinib resistance [21]. Our findings suggest that the p.(G1202R) variant is a resistant mutation in the post-alectinib treatment sample of ALK79, in addition to the p.(L1196M) mutation observed in routine diagnosis. The p.(L1196M) induces resistance to alectinib and ceritinib, and probably also to lorlatinib [23]. In ALK95, one could expect the p.(L1196M) mutation positive clone observed in ALK95 to grow out to a dominant resistant clone. The p.(C1156Y) mutation observed in the same tissue biopsy is sensitive to alectinib, while responses to other ALKi are conflicting. The disappearance of the p.(C1156Y) mutation upon treatment with alectinib is consistent with the reported sensitivity to alectinib [23]. In three other cases where a resistant mutation was found (ALK14, ALK56, and ALK60), the patients had been treated with ALKi to which those mutations are sensitive, so they are unlikely to induce resistance.

The early identification of on-target ALKi resistance mutations is critical to enable selection of the most optimal subsequent line of therapy [11,26]. We observed ALKi-resistant mutations in both treatment-naive and relapsed samples, initially undetected by NGS. This indicates that a resistant mutation present at a very low MAF in pre-treatment samples can result in a TKI resistant relapse. Due to the limited number of cases with on-target mutations, we do not have enough power to test for statistical significance related to PFS and OS. However, in our relatively small series, we did not observe a clear difference in PFS between patients for which we did or did not identify resistance mutation in pre-treatment sample. Moreover, on-target ALKi resistance mutations were observed only in a small proportion of the relapsing patients. Since the resistance-causing mechanism is unknown in a substantial proportion of the patients, the clinical value of testing for on-target resistance mutations remains limited.

We did not observe a single case where the minor clone detected in one tissue type was observed in a relapse presenting at another location. This indicates that the emergence of the resistance-causing mutation was a late event, occurring after dissemination of tumor cells to other organs. In this respect, patient ALK95 is worth mentioning. None of the two variants that were detected by ddPCR in the treatment-naïve liver biopsy emerged as a treatment resistant mutation in the first relapse when a biopsy was taken from a different organ. However, one of them emerged as a post-treatment resistance mutation after a subsequent round of TKI, when a biopsy was again taken from the liver.

Intratumor heterogeneity may cause co-existence of multiple resistance mechanisms within a patient, or even within a single biopsy, thereby complicating the selection of the most optimal TKI [3,4,31]. For eight out of nine relapse biopsies where we detected a mutation at a low MAF using ddPCR, another on-target mutation was reported in routine diagnostics. Although this may seem to be contradictory, co-occurrence of more than one on-target ALKi resistance mutation was observed in 29% (9 out of 31) of the relapse biopsies included in our study. For instance, in the third biopsy of ALK11, two ALKi resistance mutations were detected by NGS. Nonetheless, we also detected a third putative resistance mutation (p.(E1210K)), which appeared to be the dominant resistance mutation in a subsequent biopsy. Thus, despite having potential resistance clones before the start of ALKi, it is hard to predict whether this mutation will indeed present as the resistance-causing variant in subsequent relapses, especially when presenting at other sites.

Our study encountered several limitations, including the small cohort size, variability in location of analyzed biopsies, and differences in ALKi therapies administered. Additionally, intratumor heterogeneity might have led to bias into our data, as we only analyzed relatively small biopsies, which may not reflect the total tumor mass. With our goal of analyzing 6000 filled droplets, we in theory analyzed 3000 cells. It might very well be that a potential existing resistant clone was missed due to low abundance or not analyzing sufficient material. Using macrodissection, we aimed at a minimal amount of 20% tumor cells. Although still suboptimal, this was, for some of the cases, the maximum percentage that could be recovered. In an ideal setting, multiple biopsies, covering all progressive tumor locations, should be taken and analyzed in parallel. However, this is frequently not feasible. An alternative approach to circumvent intra-tumor heterogeneity could involve analyzing circulating tumor (ct)DNA isolated from blood plasma by either ddPCR or NGS-based approaches [27,32,33,34]. Although this method also has limitations, including the limit of detection, the extent to which all tumor foci contribute to the ctDNA and the amount of available cfDNA, NGS-based approaches on cfDNA have clear advantages. One main advantage is that multiple targets can be analyzed in a single experiment, enhancing sensitivity over ddPCR tests for single mutations. In a direct comparison of six different cfDNA NGS platforms, the detection rate for variants with a VAF of 1% was 100% for the four best-performing commercially available mutation analysis kits [35]. Performance dropped significantly at VAF of 0.125, with 85% and 60% detection rates for the two best performing kits. Therefore, implementation of NGS-based tests might improve the accuracy of molecular profiling and more optimally guide treatment decisions for ALK-positive NSCLC patients. Potentially, a combination of analyzing both tissue and ctDNA might be most optimal to predict on-target resistance mechanisms. A final limitation of our study is the availability of a limited number of patients, which precluded drawing of firm conclusions. Therefore, findings in this paper should be interpreted with caution and need to be validated before considering broader clinical applications.

## 5. Conclusions

Our study revealed the presence of minor clones with on-target resistant mutations in a subset of both treatment-naive and relapse biopsies from ALK-positive NSCLC patients, using the highly sensitive ddPCR technique. Notably, part of the initially observed mutations emerged as the dominant resistant mutation in the relapse tumor, but only when the relapse presented at the same location as the original biopsy was taken.

## Figures and Tables

**Table 1:** Overview of the clinical characteristics of the 17 ALK-fusion gene positive NSCLC patients presenting with ALK on-target resistance mutations following ALKi.

Patient Characteristics	Number of Patients
Male/Female	4/13
Median Age (range)	64 (34–74)
Smoking history	
Never	13
Former	3
Unknown	1
ALK fusion	
EML4:ALK20	9
IHC or FISH positive	8
Initial stage at diagnosis	
IIIA	2
IIIB	1
IVA	4
IVB	10

## Data Availability

The original contributions presented in this study are included in the article/Supplementary Material. Further inquiries can be directed to the corresponding author(s).

## Supplementary Materials

The following supporting data can be downloaded at https://www.mdpi.com/article/10.3390/cancers17071090/s1, Table S1: Overview of biopsy procurement method; Table S2: Overview of FFPE control samples used to determine cut-off values for the ddPCR assays; Table S3: Primer pairs used to generate positive controls for the indicated ALKi resistance mutations; Table S4: Gene blocks used as positive controls for three ALK resistant mutations; Table S5: Overview of the consistency between the diagnostics NGS test result and the ddPCR; Table S6: Overview of the ddPCR assays and the established cut-off values based on control FFPE tissue samples; Table S7: Overview of VAF as determined by molecular diagnostics test or by the ddPCR assay; Table S8: Overview of the total filled droplets obtained for each ddPCR assay for all patient samples; Table S9: Overview of ddPCR positive pre-treatment biopsies for which also a relapse biopsy was available; Figure S1: Schematic presentation of the production of ALK positive control DNA fragments; Figure S2: Schematic representation of the set-up of this study; Figure S3: ddPCR data of the set of FFPE controls used to determine cut-off values in the Grubb’s test.
