# Supplementary material for: Presence of On-Target Resistant Mutation in Pre-Treatment Samples of ALK Fusion Gene Positive Lung Cancer Patients
Source: Cancers (Basel). 2025 Mar 25;17(7):1090. doi: 10.3390/cancers17071090 (PMC11987820; doi:10.3390/cancers17071090)
Supplement: Supplementary file 1 [file cancers-17-01090-s001.zip › cancers-3479300-supplementary.pdf]

SUPPLEMENTAL INFORMATION

Presence of On-Target Resistant Mutation in Pre-Treatment Samples of ALK Fusion Gene Positive Lung Cancer Patients

Weiting Li [1], Fenneke Zwierenga [2], Katarina D. Andini [3], Justyna M. Bucher [3], Frank Scherpen [1], T. Jeroen N. Hiltermann [2], Harry J. M. Groen [2], Anthonie J. van der Wekken [2], Klaas Kok [3,*] and Anke van den Berg [1,*]

[1] Department of Pathology and Medical Biology, University of Groningen, University Medical Centre Groningen, 9713 GZ Groningen, The Netherlands; w.li@umcg.nl (W.L.); f.j.g.scherpen@umcg.nl (F.S.)
[2] Department of Pulmonary Diseases, University of Groningen, University Medical Centre Groningen, 9713 GZ Groningen, The Netherlands; f.zwierenga@umcg.nl (F.Z.); t.j.n.hiltermann@umcg.nl (T.J.N.H.); h.j.m.groen@umcg.nl (H.J.M.G.); a.j.van.der.wekken@umcg.nl (A.J.v.d.W.)
[3] Department of Genetics, University of Groningen, University Medical Centre Groningen, 9700 RB Groningen, The Netherlands; k.d.andini@umcg.nl (K.D.A.); justyna.bucher@gmail.com (J.M.B.)
* Correspondence: k.kok@umcg.nl (K.K.); a.van.den.berg01@umcg.nl (A.v.d.B.)

MATERIALS AND METHODS

Patient Selection, Tumor and Control Samples

All *ALK* fusion gene and mutation tests were done according to standard procedures at the time of diagnosis in our ISO15189 certified diagnostic lab. An overview of the diagnostics tests per sample is given in **Supplementary Table S1**. *ALK* translocation testing was initially performed using immunohistochemistry (IHC), fluorescence in situ hybridization (FISH) or Archer analysis. In part of the patient samples multiple techniques were applied simultaneously. Mutation analysis in the diagnostics was performed using an amplicon based IonTorrent, single-molecule molecular inversion probes (smMIP) [1], or the TSO500 according to the instructions of the manufacturers. Gene panels for these three tests are available at (https://www.umcg.nl/-/afdeling/pathologie/moleculaire-diagnostiek/next-generation-sequence-mutatieanalyse and https://www.palga.nl/voor-pathologen/moleculaire-bepaling). DNA samples isolated from formalin-fixed paraffin-embedded (FFPE) material using standard diagnostic procedures (QIAmp DNA FFPE tissue kit,Qiagen, Hilden, Germany) were retrieved from the molecular diagnostics archive. DNA extraction was performed from macrodissected tissue areas containing a tumor cell percentage of 20% or higher, as indicated by an accredited pathologist (estimates of the enriched tumor cell content are given in **supplementary Table S1**). FFPE control samples were selected from the diagnostic archive isolated in the same weeks as the ALK patient samples (For details see **supplemental Table S2**). These control FFPE samples were used to determine the cutoff value for each ddPCR assay. The final control set consisted of 17 samples, including 1 glioma, 1 colon cancer, 1 melanoma, and 14 lung cancers. All lung cancer samples were tested for ALK in the diagnostics with a negative result (by either immunohistochemistry, FISH or by Archer). Seven of the lung cancers had a *KRAS* mutation and one had an *EGFR* mutation. Overall, 11 of the 17 control cases had a known driver gene mutation with a mutant allele frequency ranging between 22% and 76% and a median of 34%. This indicates presence of sufficient tumor cells in the control FFPE blocks.

As our previous study using ddPCR on FFPE samples showed a higher number of positive droplets for DNA samples from aged as compared to freshly prepared FFPE blocks [2], we checked the time interval between the generation of FFPE blocks and the date of DNA isolation for FFPE blocks included in this study. All DNA isolations were conducted within one month of biopsy collection, ensuring a consistent age of the FFPE blocks at the time of DNA isolation for controls and patient samples. Additionally, high quality genomic DNA samples isolated from white blood cells of 10 healthy controls were used to test specificity of the mutant and wild type probes in the droplet digital PCR (ddPCR).

Generation of positive controls for ddPCR

To generate positive control DNA fragments, we subsequently conducted three PCR reactions. The first two PCR reactions utilized (a) the forward primer containing the mutation paired with a downstream primer, and (b) the reverse primer containing the mutation paired with an upstream primer. This resulted in two partially overlapping PCR fragments, each containing the mutation (**Supplementary Figure S1**). Next, a third PCR was performed using DNA from the two PCR products as templates, combined with the two flanking primers, to generate and amplify the full-length DNA fragment containing the mutation in the center. The resulting PCR products were analyzed on a 1% agarose gel, and the presence of the mutation was confirmed by Sanger sequencing (**Supplementary Table S3**). For the p.(S1206A), p.(E1210K) and p.(F1174L) mutations, gBlocks containing the specific mutation were ordered from IDT (IDT, Leuven, Belgium) (**Supplementary Table S4**). All positive control fragments were mixed with fragments lacking mutations to achieve a variant allele frequency (VAF) of approximately 15%.

Droplet Digital (dd)PCR

Droplets were generated by the ddPCR generator (Biorad, Hercules, CA, USA). The amplification steps consisted of an initial denaturation at 95°C for 10 minutes, followed by 39 cycles of denaturation at 94°C for 30 seconds, annealing at 55°C for 1 minute and a final step at 98°C for 10 minutes, followed by a cooling down step to 4°C. Fluorescence signals from individual droplets were detected by the QX200 droplet reader and analyzed using QuantaSoft Analysis Pro software (Biorad, Hercules, CA, USA). Optimal annealing temperatures were defined as the lowest temperature that yielded robust signal intensities for both wild-type and mutation-positive control DNA samples, with clear separation between the two signals.

For each assay we first performed a temperature range to determine the most optimal annealing temperature using the synthetic controls. Next, we assessed the specificity of the assays in two ways. First, we tested each ddPCR assays on genomic DNA isolated from white blood cells. Next, all assays were analyzed with all 10 synthetic positive controls to determine cross-reactivity, which is critical specifically for ALKi resistance mutations at the same or closely flanking genomic positions. Each DNA sample was analyzed in at least two independent experiments and in two wells per experiment. In general, we generated more than 15,000 droplets

per well and we aimed at having around 1,500 filled droplets per well (**Supplementary Table S2** shows the total number of filled droplets per assay and per sample). We aimed to achieve a minimum of 6,000 filled droplets for each sample. In case we obtained <6,000 filled droplets, we conducted a third experiment. All samples were analyzed for up to 10 mutations, depending on the available amount of DNA. To avoid the risk of false positive results due to cross-contamination, we initially excluded samples which were previously tested positive in the diagnostic setting with in general higher mutant allele frequency (MAF). For the same reason we diluted our synthetic positive control with wild type DNA to obtain a low MAF (about 15%) and used a low DNA input aiming to obtained around 500 filled droplets for these samples. We followed a strict protocol including working in a clean pre-PCR room to avoid contamination with the positive control or patient samples with known mutations and used positive and negative DNA control samples, along with a DNA-free sample in each experiment. For patient samples with a known ALK on-target resistance mutation for which we had sufficient DNA after doing the ddPCR assays for which we wanted to identify potential minor clones, we also did ddPCR for the known mutation to compare diagnostic test results with ddPCR results.

RESULTS

Positive controls and cut-off values per assay

After confirmation of the presence of the 10 selected *ALK* mutations in the generated positive control samples, we determined the optimal annealing temperature by performing a temperature gradient test ranging from 50 to 60°C. Based on the observed plots we determined that 55°C was suitable for all assays. Next, we assessed the specificity of the mutant probes on high quality genomic DNA. This analysis revealed a median number of positive droplets of 1 (range 0 to 8), and a median MAF of 0 (range 0 to 0.06%). To determine potential cross-reactivity between different mutations we tested all positive controls with all ddPCR assays. This revealed no cross-reactivity for 8 of the 10 assays. Limited cross-reactivity was observed for p.(L1196M) and p.(L1196Q) with much lower signals (<90%). For p.(F1174L) and p.(F1174V) we observed slightly more cross-reactivity, with signals that were <50% of the positive control. For both assays, we regarded the specificity as high enough to reliably analyze the patient samples. For 10 samples we did have sufficient DNA to validate the NGS result by ddPCR. We confirmed presence of the mutations in all samples with at similar MAF (**Supplementary Table S5**).

To establish cutoff values for the MAF per assay, we analyzed DNA samples isolated from control tumor FFPE blocks for each assay. Considering the variability observed in the MAF (medium 0.08% with a range of 0.02% to 0.39%) across the 10 mutations, we implemented individual cutoffs for each mutation to mitigate the risk of false positives using the Grubbs' critical value (**Supplementary Table S6**). Notably, C>T mutations exhibited higher cut-off values (3 assays, ranging from 0.14% to 0.19%) compared to 6 out of 7 non-C>T mutations (ranging from 0.02% to 0.1%), with one exception (**Figure 2** and **Supplementary Figure**

S3). The p.(S1206A) ddPCR assay displayed the highest cutoff value (0.39%), surpassing even the cut-off values for the C>T ddPCR assays.

Supplementary Table S1. Overview of biopsy procurement method, estimated tumor cell content of the enriched tissue areas and diagnostic tests used to analyze all patient samples included in this study.

Patient	Sample	Method of biopsy procurement	Estimated tumor %	NGS approach	ALK test
ALK56	1	EBUS	<20%	no	FISH
	2	EBUS (25 ml)	90%	IT	IHC
	3	EBUS (15ml)	90%	smMIP	IHC
ALK 95	1	Needle biopsy	80%	IT	IHC
	2	CT guided biopsy (1,6 cm, 16G)	30%	smMIP	IHC
	3	Needle biopsy (4,5 cm, 18G)	40%	smMIP	nS
	4	Needle biopsy (3,6 cm, 18G)	60%	TSO500	Archer
ALK58	1	Needle biopsy (1,6 cm)	60%	IT	FISH
	2	Needle biopsy (1 cm)	30%	IT	FISH
ALK4	1	Surgical organ resection (total)	n.a.	no	FISH
	2	Surgical biopsy (3,5 x 3,5 x 2 cm)	n.a.	no	FISH
	3	Needle biopsy (4,7 cm)	70%	IT	IHC
	4	Needle biopsy (4 cm)	30%	IT	IHC
ALK14	1	Needle biopsy	n.a.	no	n.a.
	2	Surgical biopsy (2 x 1,3 x 0,5 cm)	n.a.	no	FISH and IHC
ALK54	1	Needle biopsy	n.a.	no	IHC and FISH
	2	Needle biopsy	60%	IT	nS
ALK11	1	EBUS	n.a.	no	FISH
	2	EBUS	50%	IT	FISH
	3	EBUS	20%	IT	FISH
	4	EUS (30ml)	20%	IT	IHC
ALK32	1	Needle biopsy (2 cm)	n.a.	IT	FISH
	2	Needle biopsy (4 cm)	80%	IT	IHC
	3	Needle biopsy (7 cm)	70%	IT	IHC
	4	Needle biopsy (3,5 cm)	50%	IT	IHC and nS
ALK60	1	Needle biopsy	n.a.	IT	FISH
	2	Needle biopsy (2,1 cm)	70-80%	IT	IHC and nS
	3	Needle biopsy (1,6 cm)	70%	smMIP	nS
	4	Needle biopsy (4,2 cm)	70%	smMIP	nS
ALK57	1	Pleural effusion cytology	50%	IT	IHC
	2	Needle biopsy (1,2 cm)	60%	smMIP	IHC
	3	Needle biopsy (0,6 cm)	50%	smMIP	nS
	4	Needle biopsy	50%	IT	Archer
ALK61	1	Pleural effusion cytology	50%	IT	IHC
	2	Pleural effusion cytology	20%	IT	nS
ALK55	1	Needle biopsy (1,2 cm)	70%	IT	IHC
	2	Needle biopsy (2,5 cm)	60%	IT	IHC
	3	Needle biopsy (1,8 cm)	60%	IT	IHC

ALK53	1	Pleural effusion cytology	n.a.	IT	IHC
	2	Pleural effusion cytology	20%	IT	IHC
ALK59	1	Pleural effusion cytology	70%	IT	FISH
	2	EUS (15ml)	50%	IT	n.a.
ALK79	1	Surgical biopsy (1 x0,7 x 0,5 cm)	30%	IT	nS
	2	Pleural effusion cytology	80%	smMIP	IHC and nS
ALK51	1	Surgical biopsy	80%	IT	IHC
	2	Surgical biopsy (4,5 x 2,5 x 0,2 cm)	40%	smMIP	IHC and nS
	3	Needle biopsy (3,6 cm)	60%	smMIP	Archer
ALK96	1	EBUS	60%	IT	Archer
	2	EUS (30ml)	30%	TSO500	Archer

EBUS: endo bronchial ultrasound: a (small) needle biopsy through the airway, guided through ultrasound in the airway. EUS: endoscopic ultrasound: a (small) needle biopsy through the airway, guided through ultrasound in the esophagus. CT guided biopsy: a needle biopsy through the skin, guided via CT scan. Needle biopsy: a needle biopsy through the skin, guided through ultrasound on the skin at the target organ. Surgical biopsy or total organ resection. Pleural effusion cytology: embedded cells from fluid. IT: Ion Torrent™ technology; SmMIP: use single-molecule molecular inversion probes (smMIP). TSO500: Targeted sequencing of DNA from 523 genes. FISH: Fluorescence in situ hybridization (FISH); IHC: Immunohistochemistry; nS: NanoString; Archer: RNA based fusion detection system. n.a., not available.

Supplementary Table S2. Overview of FFPE control samples used to determine cutoff values for the ddPCR assays.

Sample	Tumor type	Genomic aberrations	MAF (%)
C18-1	lung cancer	no ALK fusion and no other driver gene mutations or fusions	
C19-1	lung cancer	no ALK fusion and no other driver gene mutations or fusions	
C19-2	lung cancer	no ALK fusion and no other driver gene mutations or fusions	
C19-3	lung cancer	no ALK fusion and no other driver gene mutations	
C19-4	lung cancer	no ALK or other fusion genes; KRAS p.(G12D)	34%
C20-1	lung cancer	no ALK or other fusion genes; KRAS p.(G12C)	76%
T17-3	glioma	IDH1 mutation p.(R132H)	33%
T17-4	lung cancer	no ALK or other fusion genes; KRAS p.(G12D)	32%
T17-5	lung cancer	no ALK or other fusion genes; KRAS p.(G12V)	27%
T17-6	lung cancer	no ALK or other fusion genes; KRAS p.(G12D)	22%
T17-7	lung cancer	no ALK or other fusion genes; EGFR exon19del	49%
T18-1	colon cancer	BRAF p.(V600E)	41%
T19-1	lung cancer	no ALK or other fusion genes; KRAS p.(G13C)	65%
T19-2	lung cancer	no ALK fusion gene and no other driver gene mutations	
T20-2	lung cancer	no ALK or other fusion genes; p.(KRAS G12S)	33%
T20-3	melanoma	NRAS p.(Q61L)	41%
T20-4	lung cancer	no ALK fusion and no other driver gene mutations or fusions	

Supplementary Table S3. Primer pairs used to generate positive controls for the indicated ALKi resistance mutations.

c.3806G>C (p.G1269A) (chr2:29209816)(1)	
Upstream	5'-AAGGTTTCCCATAGCCTGAAAAGG-3'
Reverse	5'-GTGGCCAAGATTGCAGACTTCGGGATG-3'
Forward	5'-CATCCCGAAGTCTGCAATCTTGGCCAC-3'
Downstream	5'-AGGCTGTTTCTCTCACACTGAAGTA-3'
c.3604G>A (p.G1202R) (chr2:29220747)(1)	
Upstream	5'-TTGCTACCCAGGCTGCCCAC-3'
Reverse	5'-GAGCTCATGGCGGGGAGAGACCTCAAG-3'
Forward	5'-CTTGAGGTCTCTCCCCGCCATGAGCTC-3'
Downstream	5'-TGTGGTTCTTCCCACCTGCTCAC--3'
c.3587T>A (p.L1196Q) (chr2:29220764)(1)	
Upstream	5'-TTCTCTTCCAGCCAGTCAGTCAC-3'
Reverse	5'-CCGGTTCATCCTGCAGGAGCTCATGGC-3'
Forward	5'-GCCATGAGCTCCTGCAGGATGAACCGG-3'
Downstream	5'-CCAGCAAGATTCTGGGTTTAGGCT-3'
c.3586C>A (p.L1196M) (chr2:29220765)(1)	
Upstream	5'-TTCTCTTCCAGCCAGTCAGTCAC-3'
Reverse	5'-CCGGTTCATCCTGATGGAGCTCATGGC-3'
Forward	5'-GCCATGAGCTCCATCAGGATGAACCGG-3'
Downstream	5'-CCAGCAAGATTCTGGGTTTAGGCT-3'
c.3467G>A (p.C1156Y) (chr2:29222392)(1)	
Upstream	5'-GGGGACATGCTAGGGACAACAC-3'
Reverse	5'-CTGCCTGAAGTGTACTCTGAACAGGAC-3'
Forward	5'-GTCCTGTTCAGAGTACACTTCAGGCAG-3
downstream	5'-GGCTTGCGGACTCTGTAGGCT-3'
c.3512T>A (p.I1171N) (chr2:29222347)(1)	
Upstream	5'-TGGAGAAAAGGGGACATGCTAGGG-3'
Reverse	5'-GAAGCCCTGATCAACAGGTAAAGCCACAGAG -3'
Forward	5'-CTCTGTGGCTTTACCTGTTGATCAGGGCTTC -3'
Downstream	5'-CCCTGCAAGTGGCTGTGAAGGTAAG-3'
c.3520T>G (p.F1174V) (chr2:29220831)(1)	
Upstream	5'-TTGCTACCCAGGCTGCCCAC-3'
Reverse	5'-CTGCTCTGCAGCAAAGTCAACCACCAGAAC-3'
Forward	5'-GTTCTGGTGGTTGACTTTGCTGCAGAGCAG-3'
Downstream	5'-TGTGGTTCTTCCCACCTGCTCAC-3'

(1)genomic locations according to HG38

Supplementary Table S4. Gene blocks used as positive controls for three ALK resistant mutations.

c.3520T>C p.(F1174L) (chr2:29220831)(1)
AGGAAGGACTTGAGGTCTCCCCCCGCCATGAGCTCCAGCAGGATGAACCGGGGCAGGGATTGCAGGCTCACCCC<br>AATGCAGCGAACAATGTTCTGGTGGTTGAGTTTGCTGCAGAGCAGAGAGGGATGTAACCAAAATTAACTGAGCT<br>GAGTCTGGGCAAATCTTAAACTGGGAGGAACAGGATACAAAGTTACATTTTCAGCAGCTACAATGTATA
c.3616T>G (p.S1206A) (chr2:29220735)(1)
GCCCACTCTTGCTCCTTCCATCCTTGCTCCTGTCCTTGGCACAACAACTGCAGCAAAGACTGGTTCTCACTCACCG<br>GGCGAGGGCGGGTCTCTCGGAGGAAGGCCTTGAGGTCTCCCCCCGCCATGAGCTCCAGCAGGATGAACCGGGGC<br>AGGGATTGCAGGCTCACCCCAATGCAGCGAACAATGTTCTGGTGGTTGAATTTGCTGCAGAGCAGAGA
c.3628G>A (p.E1210K)(chr2:29220723)(1)
GCTACCCAGGCTGCCCACTCTTGCTCCTTCCATCCTTGCTCCTGTCCTTGGCACAACAACTGCAGCAAAGACTGGT<br>TCTCACTCACCGGGCGAGGGCGGGTCTTTCGGAGGAAGGACTTGAGGTCTCCCCCCGCCATGAGCTCCAGCAGG<br>ATGAACCGGGGCAGGGATTGCAGGCTCACCCCAATGCAGCGAACAATGTTCTGGTGGTTGAATTTGCTGC

(1)genomic locations according to HG38

Supplementary Table S5. Overview of the consistency between the diagnostics NGS test result and the ddPCR.

Assay	Sample	VAF ddPCR	VAF NGS report
c.3604G>A p.(G1202R)	ALK96-2	12%	12%
c.3586C>A p.(L1196M)	ALK32-4	13%	13%
c.3520T>G p.(F1174V)	ALK32-3	17%	19%
c.3520T>G p.(F1174V)	ALK32-4	23%	23%
c.3467G>A p.(C1156Y)	ALK61-2	44%	43%
c.3806G>C p.(G1269A)	ALK51-2	62%	61%
c.3806G>C p.(G1269A)	ALK51-3	62%	61%
c.3806G>C p.(G1269A)	ALK59-2	20%	15%
c.3616T>G p.(S1206A)	ALK11-4	15%	14%
c.3628G>A p.(E1210K)	ALK11-4	14%	13%

Supplementary Table S6. Overview of the ddPCR assays and the established cut-off values based on control FFPE tissue samples.

ALK mutation	Biorad Assay ID	Grubb's test based cut-off value	Average droplets generated (StDev) (*)	Percentage filled droplets (StDev)(*)
c.3806G>C p.(G1269A)	dHsaMDV2010081	0.06%	14,753 (4064)	14 (6)
c.3616T>G p.(S1206A)	dHsaMDS287775170	0.39%	15,553 (4243)	16 (7)
c.3604G>A p.(G1202R)	dHsaMDS744393308	0.19%	11,397 (2950)	14 (8)
c.3587T>A p.(L1196Q)	dHsaMDS958969903	0.06%	15,055 (3559)	12 (6)
c.3586C>A p.(L1196M)	dHsaMDS468586747	0.03%	13,717 (3612)	17 (12)
c.3520T>G p.(F1174V)	dHsaMDS2514636	0.04%	13,869 (3679)	14 (7)
c.3520T>C p.(F1174L)	dHsaMDS75304011	0.10%	13,751 (2795)	22 (7)
c.3512T>A p.(I1171N)	dHsaMDS300396723	0.02%	15,252 (3386)	13 (6)
c.3467G>A p.(C1156Y)	dHsaMDS71891584	0.16%	13,222 (2308)	18 (9)
c.3628G>A p.(E1210K)	dHsaMDS729733181	0.14%	14,585 (2500)	20 (7)

(*) based on analysis of patient samples.

Supplementary Table S7. Overview of VAF as determined by molecular diagnostics test or by the ddPCR assay.

Patient	Sample	p.(G1202R)	p.(C1156Y)	p.(L1196M)	p.(L1196Q)	p.(F1174V)	p.(F1174L)	p.(G1269A)	p.(I1171N)	p.(S1206A)	p.(E1210K)
ALK59	1	—	—	—	—	—	—	—	—	—	—
	2	—	—	—	—	—	—	15	—	—	—
ALK53	1	—	—	—	—	—	—	—	—	—	—
	2							3	5		
ALK58	1	—	—	—	—	—	—	—	—	—	—
	2			34	—	—		0.066	—		
ALK55	1	—	—	—	—	—	—	—	—	—	—
	2			11							
	3			25							
ALK96	2	12	—	—	—	—	10	—	—	—	—
ALK61	1	—	—	—	—	—		—	—	—	
	2	—	43	—	—	—	—	—	—	—	—
ALK79	1	—	—	—	—	—	—	—	—	—	—
	2	2.679	—	50	—	—	—	—	—	—	—
ALK51	1	—	—	—	—	—	—	—	—	—	—
	2	—	—	—	—	—	—	61	—	—	—
	3			0.076	—	—	—	61	—	—	—
ALK95	1	—	0.177	0.089	—	—	—	—	—	—	—
	2	—		—	—	—		—			
	3			15			7				
	4	11		11	—	—	—	—	—	—	—
ALK60	1	—	—	—	—	—		—	—	0.503	
	2	35		—	—	—		—	—		
	3	—		—	—	—		—			
	4	—							—		
ALK32	1	—	—	—	—	—	—	—	—	—	—
	2			—	18	—		—	—		
	3			0.089	—	19	—	—	—	—	—
	4	—	—	13	—	23	—	0.083	—	—	—
ALK57	1	—	—	—	—	—	—	—	—	—	—
	2	—	—	0.075		—	—	—			—
	3	20	—	0.062	—		—	0.1	—	—	—
	4	46	13	—	—	—		18		—	
ALK11	2	—								21	
	3	33	—				—			34	0.141
	4	—	—	—	—	—	—	—	—	14	13
ALK56	1	—		0.087				—			
	2	5		—	—	—			—		
	3	49					—			—	—
ALK54	1										
	2	—	—	20	—	—	—	—	—	—	—
ALK4	3	—	15	0.131	—						
	4	13	—	—	—	—	—	10	—	—	—
ALK14	1	—									0.215
	2	20	—	—	—	—		—	—		

-, ddPCR test was negative based on our criteria; VAF shown in black is based on molecular diagnostic NGS result; VAF shown in red is based on ddPCR result.

Supplementary Table S8. Overview of the total filled droplets obtained for each ddPCR assay for all patient samples.

Patient	Sample	p.(G1202R)	p.(C1156Y)	p.(L1196M)	p.(L1196Q)	p.(F1174V)	p.(F1174L)	p.(G1269A)	p.(I1171N)	p.(S1206A)	p.(E1210K)
ALK59	1	10087	7861	13374	6511	9735	7943	11036	8188	8618	12374
	2	6787	13426	9358	7077	8898	6799	78571	8033	8497	6634
ALK53	1	8685	6532	7701	8275	7683	7824	8482	7552	6228	9178
	2										
ALK58	1	6428	7658	7732	7782	6873	7347	7025	7699	7100	8020
	2				5277	7157		5793	7992		
ALK55	1	8064	9351	8448	6411	7497	8963	6638	5796	8186	10827
	2										
	3										
ALK96	2	24950	10941	10921	14443	6268		7901	9074	7437	7487
ALK61	1	7888	6322		6104	7204		6697	6982	7232	
	2	9635	46277	23831	8847	6971	12676	9274	7297	6521	7270
ALK79	1	6872	6474	8353	7174	7388	7950	7457	6469	7999	9712
	2	5964	16154		5764	7703	10603	8743	10217	8274	7017
ALK51	1	10782	6903	7318	9282	6715	10133	6025	7269	7595	6331
	2	17955	26505	9852	9328	7579	8180	47772	6283	8364	7995
	3			8053	8827	16179	16909	16862	12872	9683	7192
ALK95	1	6131	7336	7442	6901	6700	15055	6680	6827	7059	9921
	2	11262		4810	7032	5500		3614			
	3										
	4				9280	5945	8350	6356	7524	7956	6574
ALK60	1	8542	8442	6294	7591	10259		10739	7817	4036	
	2			5393	7061	5220		7857	157		
	3	7729		4806	4616	4852		1316			
	4	6219							5379		
ALK32	1	4872		19009	9436	6272	6794	13053	9401	8583	8217
	2							2540	5604		
	3			10234	7831	38035	9681	7133	7680	11526	8040
	4	9568	7838		8151	21618	6638	8790	8083	10364	8613
ALK57	1	9142	6869	7525	6955	11692	7544	9584	7047	8491	7716
	2	7536		13433			8093	5376			8565
	3		11007	8833	7011	8753	9631	9196	8365	6683	7624
	4			3198	604	612				1820	
ALK11	2	3789									
	3		9021				8841				8373
	4	10687	11288	7232	7391	8909	7455	8101	5861	17554	16786
ALK56	1	7654		7628				7156			
	2			5479	6495	6331		7151	1172		
	3						7044			9156	6761
ALK54	1										
	2	7584	7850		8124		8862	7667	9415	8258	8087
ALK4	3	3067		5899	5326						
	4		9616	10401	5729	6780	8628	6909	7434	9021	6810
ALK14	1										3188
	2		7820	6356	6472	5813		8589	6768		

Yellow highlights are tests (n=7) for which we did have <3,000 filled droplets, so these results are ambiguous and might be false negative. Green highlights (n=11) are tests for which the total number of filled droplets were between 3,000 and 5,000, although we did not reach the detection limit of 0.1%, we still regard them as most likely negative in our study. Numbers shown in red are ddPCR positive samples and numbers shown in black are ddPCR negative cases.

Supplementary Table S9. Overview of ddPCR positive pre-treatment biopsies for which also a relapse biopsy was available.

Case	pre-treatment biopsy			TKI treatment	relapse biopsy			PFS (months)
	sample	tissue	variant by ddPCR		sample	tissue	MAF (%) by NGS	
**Treatment-naive samples**								
ALK95	1	liver	p.(C1156Y)	Alectinib	2	muscle	nd	19.4
			p.(L1196M)				nd	
ALK56	1	lympoh node	p.(L1196M)	crizotinib	2	bronchy	nd	13.9
ALK14	1	muscle	p.(E1210K)	crizotinib	2	lymph node	nd	8.2
**Relapse samples**								
ALK32	3	liver	p.(F1174V)	lorlatinib	4	liver	13	13.9
ALK57	2	muscle	p.(L1196M)	alectinib	3	lung	nd	8.4
ALK57	3	lung	p.(L1196M)	lorlatinib	4	lung	nd	21.2
			P.(G1269A)				18	
ALK11	3	lymph node	p.(E1210K)	alectinib	4	lymph node	13	3.5
ALK4	3	liver	P.(L1196M)	alectinib	4	fatty	nd	19.1

nd: not detected; Text shown in red indicates patients for which pre- and post-treatment biopsies originated from the same location.

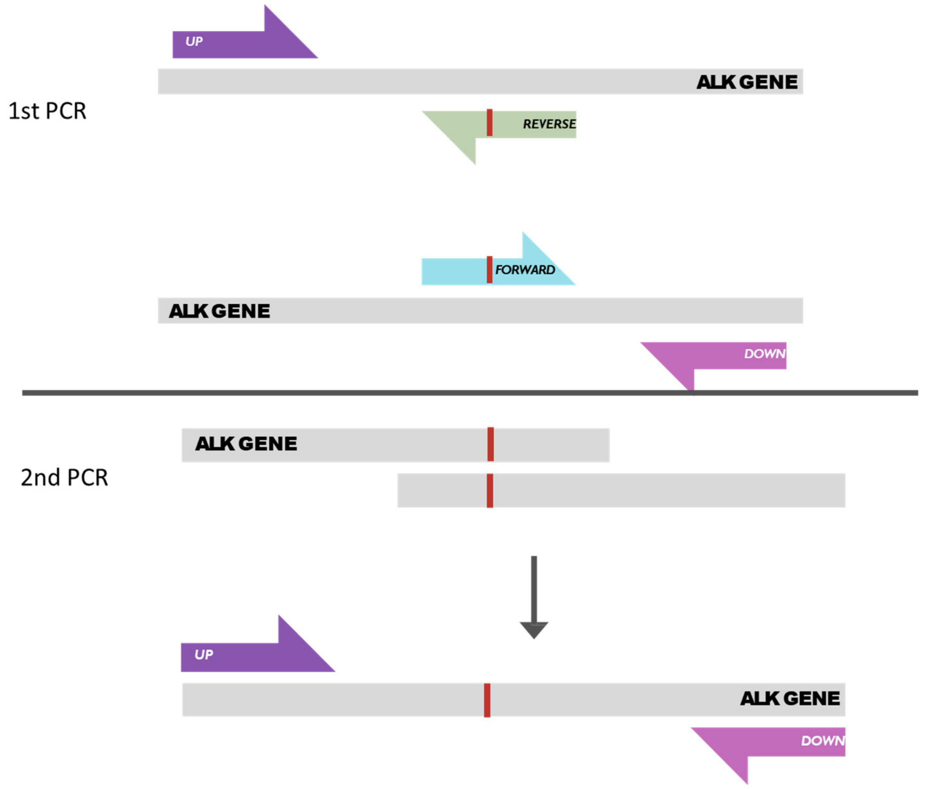

Supplementary Figure S1. Schematic presentation of the production of *ALK* positive control DNA fragments. For each *ALK* point mutation (p.(G1202R), p.(L1196M), p.(L1196Q), p.(F1174V), p.(C1156Y), p.(G1269A), p.(I1171N), four primers were designed (IDT, Leuven, Belgium) (Supplementary Table S1). These primers included one upstream and one downstream primer flanking the mutation site, as well as two primers (a forward and a reversed primer) overlapping with the mutated nucleotide and with each other.

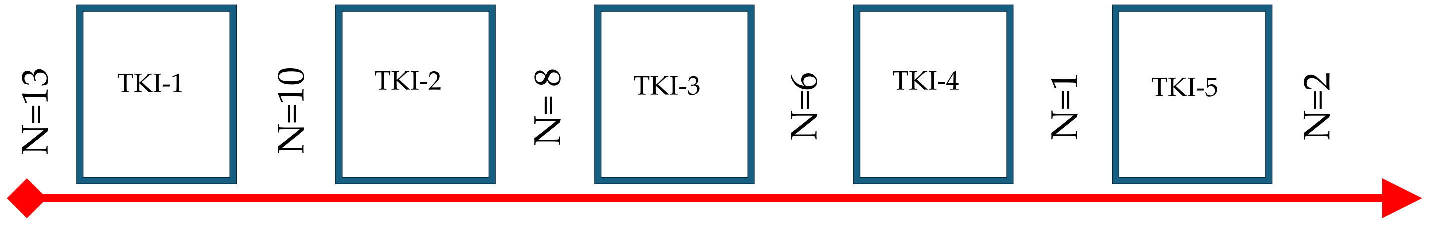

Supplementary Figure S2: Schematic representation of the set-up of this study. ALK fusion gene-positive patients are often treated successively with multiple TKIs. The number of TKIs will vary per patient. The red arrow indicates the timeline with the diamond shape indicating the time of diagnosis. The dark blue squares indicate successive treatments. As a result of a treatment patients often relapse and a new biopsy is taken. Based on the molecular findings in this new biopsy a new TKI is selected for a subsequent line of treatment. The number of samples available for this study before each line of treatment is indicated between the boxes. The leftmost number indicates the number of treatment-naïve samples. We used the biopsies before each treatment to interrogate if the resistant mutation of the following TKI is already present as a minor clone in these biopsies. Biopsies are tested for up to 10 ALK on-target variants.

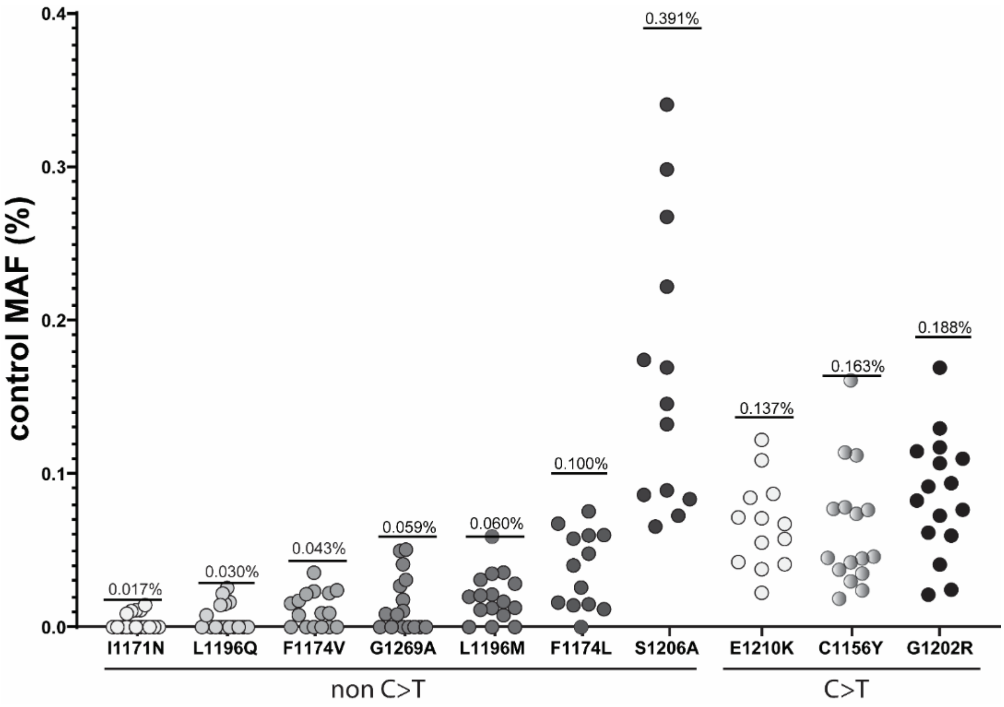

Supplementary Figure S3. ddPCR data of the set of FFPE controls used to determine cut-off values in the Grubb's test. Mutant allele frequencies for 13-16 control FFPE samples for the ALK variants included in this study. Horizontal lines show the cut-off value as determined by the Grubb’s test. The mutations are shown in two groups based on being a C>T mutation or not.

Reference list

1. Eijkelenboom A, Kamping EJ, Kastner-van Raaij AW, Hendriks-Cornelissen SJ, Neveling K, Kuiper RP, Hoischen A, Nelen MR, Ligtenberg MJ, Tops BB: **Reliable Next-Generation Sequencing of Formalin-Fixed, Paraffin-Embedded Tissue Using Single Molecule Tags**. *J Mol Diagn* 2016, **18**(6):851-863.
2. Li W, Kok K, Tan GW, Meng P, Mastik M, Rifaela N, Scherpen F, Hiltermann TJN, Groen HJM, Wekken AJV *et al*: **The Presence of EGFR T790M in TKI-Naive Lung Cancer Samples of Patients Who Developed a T790M-Positive Relapse on First or Second Generation TKI Is Rare**. *Cancers (Basel)* 2022, **14**(14).
